# Supplementary material for: A UMLS-based spell checker for natural language processing in vaccine safety
Source: BMC Med Inform Decis Mak. 2007 Feb 12;7:3. doi: 10.1186/1472-6947-7-3 (PMC1805499; doi:10.1186/1472-6947-7-3)
Supplement: Additional file 13 — RAP application directory. Sets up directory for RAP (RDF application for PHP) source code [file 1472-6947-7-3-S13.gz › rap/doc/phpdoc/__filesource/fsource_ontModel__ontModelOntClass.php.html]

xml version="1.0" encoding="iso-8859-1"?


File Source for OntClass.php


# Source for file OntClass.php

Documentation is available at OntClass.php

```
1. <?PHP
2. /**
3. * ----------------------------------------------------------------------------------
4. * Class: OntClass
5. * ----------------------------------------------------------------------------------
6. *
7. * @package     ontModel
8. */


11. /**
12. * Class that represents an ontology node characterising a class description.
13. *
14. * <BR><BR>History:
15. * <LI>10-05-2004                : First version of this class.</LI>
16. *
17. * @version  V0.9.1
18. * @author Daniel Westphal <mail at d-westphal dot de>
19. *
20. *
21. * @package     ontModel
22. * @access    public
23. ***/
24. class OntClass extends OntResource
25. {
26. /**
27. * Constructor
28. * You can supply a uri
29. *
30. * @param string $uri
31. * @access    public
32. */
33. function OntClass($uri = null)
34. {
35. parent::OntResource($uri);
36. }

38. /**
39. * Add a sub-class of this class.
40. *
41. * @param    object ResResource        $resResource
42. * @return    boolean
43. * @access    public
44. */
45. function addSubClass($resResource)
46. {
47. return $resResource->addProperty($this->vocabulary->SUB_CLASS_OF(),$this);
48. }

50. /**
51. * Add a super-class of this class.
52. *
53. * @param    object ResResource        $resResource
54. * @return    boolean
55. * @access    public
56. */
57. function addSuperClass($resResource)
58. {
59. return $this->addProperty($this->vocabulary->SUB_CLASS_OF(),$resResource);
60. }

62. /**
63. * Answer a class that is the sub-class of this class.
64. * If there is more than one such class, an arbitrary selection is made.
65. *
66. * @return    object OntClass or NULL
67. * @access    public
68. */
69. function getSubClass()
70. {
71. $statement = $this->model->findFirstMatchingStatement(null,$this->vocabulary->SUB_CLASS_OF(),$this);
72. if ($statement !== null)
73. return $this->model->createOntClass($statement->getLabelSubject());

75. return null;
76. }

78. /**
79. * Answer a class that is the super-class of this class.
80. * If there is more than one such class, an arbitrary selection is made.
81. *
82. * @return    object OntClass or NULL
83. * @access    public
84. */
85. function getSuperClass()
86. {
87. return $this->getPropertyValue($this->vocabulary->SUB_CLASS_OF(),'OntClass');
88. }

90. /**
91. * Answer true if the given class is a sub-class of this class.
92. * $direct - If true, only search the classes that are directly
93. * adjacent to this class in the class hierarchy.
94. *
95. * @param    object ResResource        $resResource
96. * @param    boolean            $direct
97. * @return    boolean
98. * @access    public
99. */
100. function hasSubclass($resResource, $direct = true)
101. {
102. if ($direct)
103. return $resResource->hasProperty($this->vocabulary->SUB_CLASS_OF(),$this);

105. $index=array();
106. return ($this->_getSubAttributeStatementsRec($this,$this->vocabulary->SUB_CLASS_OF(),$index,$resResource) === true);
107. }

109. /**
110. * Answer true if the given class is a super-class of this class.
111. * $direct - If true, only search the classes that are directly
112. * adjacent to this class in the class hierarchy.
113. *
114. * @param    object ResResource        $resResource
115. * @param    boolean            $direct
116. * @return    boolean
117. * @access    public
118. */
119. function hasSuperClass($resResource, $direct = true)
120. {
121. if ($direct)
122. return $this->hasProperty($this->vocabulary->SUB_CLASS_OF(),$resResource);

124. $index=array();
125. return ($this->_getSuperAttributeStatementsRec($this,$this->vocabulary->SUB_CLASS_OF(),$index,$resResource) === true);
126. }

128. /**
129. * Answer an ResIterator over the individuals in the model that have this class
130. * among their types.
131. *
132. * @return    object ResIterator
133. * @access    public
134. */
135. function listInstances()
136. {
137. /*
138. $statements= $this->model->find(null,$this->vocabulary->TYPE(),$this);
139. $return = array();
140. $returnIndex=array();
141. foreach ($statements as $statement)
142. {
143. $subjectLabel=$statement->getLabelSubject();
144. if (!in_array($subjectLabel,$returnIndex))
145. {
146. $returnIndex[]=$subjectLabel;
147. $return[]=$statement->getSubject();
148. }
149. }
150. return $return;
151. */
152. return new ResIterator(null,$this->vocabulary->TYPE(),$this,'s',$this->model,'Individual');

154. }

156. /**
157. * Answer an array over the classes that are declared to be sub-classes of this class.
158. * Each element of the array will be an OntClass.
159. * $direct - If true, only search the classes that are directly
160. * adjacent to this class in the class hierarchy.
161. *
162. * @param    boolean        $direct
163. * @return    array
164. * @access    public
165. */
166. function listSubClasses($direct = true)
167. {
168. $return = array();
169. if ($direct)
170. {
171. $statements = $this->model->find(null,$this->vocabulary->SUB_CLASS_OF(),$this);
172. } else
173. {
174. $index = array();
175. $statements = $this->_getSubAttributeStatementsRec($this,$this->vocabulary->SUB_CLASS_OF(),$index);
176. }

178. $returnIndex=array();
179. foreach ($statements as $statement)
180. {
181. $subjectLabel=$statement->getLabelSubject();
182. if (!in_array($subjectLabel,$returnIndex))
183. {
184. $returnIndex[]=$subjectLabel;
185. $return[]=$this->model->createOntClass($subjectLabel);
186. }
187. }
188. return $return;
189. }

191. /**
192. * Answer an array over the classes that are declared to be super-classes of this class.
193. * Each element of the array will be an OntClass.
194. * $direct - If true, only search the classes that are directly
195. * adjacent to this class in the class hierarchy.
196. *
197. * @param    boolean        $direct
198. * @return    array
199. * @access    public
200. */
201. function listSuperClasses($direct = true)
202. {
203. $return = array();
204. if ($direct)
205. return $this->listProperty($this->vocabulary->SUB_CLASS_OF(),'OntClass');

207. $index=array();
208. $statements = $this->_getSuperAttributeStatementsRec($this,$this->vocabulary->SUB_CLASS_OF(),$index);
209. $returnIndex=array();
210. foreach ($statements as $statement)
211. {
212. $objectLabel=$statement->getLabelObject();
213. if (!in_array($objectLabel,$returnIndex))
214. {
215. $returnIndex[]=$objectLabel;
216. $return[]=$this->model->createOntClass($objectLabel);
217. }
218. }
219. return $return;
220. }

222. /**
223. * Remove the given class from the sub-classes of this class.
224. *
225. * @param    object ResResource    $resResource
226. * @return    boolean
227. * @access    public
228. */
229. function removeSubClass($resResource)
230. {
231. return    $this->model->remove(new Statement($resResource,$this->vocabulary->SUB_CLASS_OF(),$this));
232. }

234. /**
235. * Remove the given class from the super-classes of this class.
236. *
237. * @param    object ResResource        $resResource
238. * @return    boolean
239. * @access    public
240. */
241. function removeSuperClass($resResource)
242. {
243. return $this->removeProperty($this->vocabulary->SUB_CLASS_OF(),$resResource);
244. }

246. /**
247. * Assert that this class is super-class of the given class.
248. * Any existing statements for subClassOf on prop will be removed.
249. *
250. * @param    object ResResource        $resResource
251. * @access    public
252. */
253. function setSubClass($resResource)
254. {
255. foreach ($this->listSubClasses() as $oldRes)
256. {
257. $this->removeSubClass($oldRes);
258. }
259. $this->addSubClass($resResource);
260. }

262. /**
263. * Assert that this class is sub-class of the given class.
264. * Any existing statements for subClassOf on prop will be removed.
265. *
266. * @param    object ResResource        $resResource
267. * @access    public
268. */
269. function setSuperClass($resResource)
270. {
271. $this->setPropertyValue($this->vocabulary->SUB_CLASS_OF(),$resResource);
272. }

274. /**
275. * Answer a resource that represents an instance of this OntClass and Individual
276. * node in this model.
277. * If a resource with the given uri exists in the model, it will be re-used.
278. * If not, a new one is created in the updateable sub-model of the ontology model.
279. *
280. * @param    string    $uri
281. * @return    object Individual
282. * @access    public
283. */
284. function createInstance($uri = null)
285. {
286. $instance = $this->model->createIndividual($uri);
287. $instance->setInstanceRdfType($this);
288. return $instance;
289. }
290. }
291. ?>
```

Documentation generated on Thu, 7 Jul 2005 13:42:20 +0200 by phpDocumentor 1.3.0RC3
